# Kinematic and Clinical Outcomes to Evaluate the Efficacy of a Multidisciplinary Intervention on Functional Mobility in Parkinson's Disease

**DOI:** 10.3389/fneur.2021.637620

**Published:** 2021-03-23

**Authors:** Raquel Bouça-Machado, Diogo Branco, Gustavo Fonseca, Raquel Fernandes, Daisy Abreu, Tiago Guerreiro, Joaquim J. Ferreira, The CNS Physiotherapy Study group

**Affiliations:** ^1^Instituto de Medicina Molecular João Lobo Antunes, Lisbon, Portugal; ^2^CNS - Campus Neurológico, Torres Vedras, Portugal; ^3^LASIGE, Faculdade de Ciências, Universidade de Lisboa, Lisbon, Portugal; ^4^Laboratory of Clinical Pharmacology and Therapeutics, Faculdade de Medicina, Universidade de Lisboa, Lisbon, Portugal

**Keywords:** Parkinson's disease, functional mobility, outcome measures, gait, sensors, digital health, wearable, technology

## Abstract

**Introduction:** Functional mobility (FM) is a concept that incorporates the capacity of a person to move independently and safely to accomplish tasks. It has been proposed as a Parkinson's disease (PD) functional and global health outcome. In this study, we aimed to identify which kinematic and clinical outcomes changes better predict FM changes when PD patients are submitted to a specialized multidisciplinary program.

**Methods:** PD patients engaged in a pre-defined specialized multidisciplinary program were assessed at admission and discharge. Change from baseline was calculated for all kinematic and clinical outcomes, and Timed Up and Go (TUG) was defined as the primary outcome for FM. A stepwise multivariate linear regression was performed to identify which outcome measures better predict TUG changes.

**Results:** Twenty-four patients were included in the study. The changes in TUG Cognitive test, supervised step length, and free-living (FL) step time asymmetry were identified as the best predictors of TUG changes. The supervised step length and FL step time asymmetry were able to detect a small to moderate effect of the intervention (*d* values ranging from −0.26 to 0.42).

**Conclusions:** Our results support the use of kinematic outcome measures to evaluate the efficacy of multidisciplinary interventions on PD FM. The TUG Cognitive, step length, and FL step time asymmetry were identified as having the ability to predict TUG changes. More studies are needed to identify the minimal clinically important difference for step length and FL step time asymmetry in response to a multidisciplinary intervention for PD FM.

## Introduction

Functional mobility (FM) in Parkinson's disease (PD) has been recently described as a person's physiological ability to move independently and safely in a variety of environments in order to accomplish functional activities or tasks and to participate in activities of daily living at home, at work, and in the community (1, 2). From the early disease stage, PD patients experience limitations in their FM. With disease progression, these limitations are usually a major cause of disability and loss of independence (1).

FM has been reported as a useful outcome measure to understand patients' overall health status, to address their daily needs related to mobility and social participation, and for monitoring, in a closer and more realistic fashion, the impact of disease progression and the effect of therapeutic interventions (2–4). The Timed Up and Go (TUG) test is a quick and easy-to-use test, specifically designed to measure FM that includes the three anchors of the concept, i.e., gait, balance, and postural transitions (2, 4, 5). Although it is the recommended tool for assessing FM in PD, other clinical tests are also used (2, 4, 5).

The development of technology-based objective measures (TOMs) and the possibility of using accurate and reliable quantitative information to evaluate PD patients' gait enable a more objective and ecological (i.e., closer to patients' real-life environment performance) perspective of patients' FM (6, 7). A recent systematic review on outcome measures for assessing FM in PD included nine studies using kinematic gait parameters (2). The authors emphasize the important role of TOMs in monitoring FM throughout disease progression. They also highlight that despite the capacity of current devices to capture large amounts of data and a great diversity of parameters, the best kinematic parameters for assessing FM in PD remain to be defined (2).

In this study, we aimed to identify which kinematic and clinical outcome measures better predict FM changes when PD patients are submitted to a specialized multidisciplinary intervention.

## Methods

### Study Design

A pragmatic prospective clinical study was conducted.

### Objective

The objective of this study is to identify the kinematic and clinical outcome measures that better predict FM changes when PD patients are submitted to a specialized multidisciplinary intervention.

### Participants

Study participants were recruited from CNS—Campus Neurológico, a tertiary specialized movement disorders center in Portugal. Patients were eligible if they had a diagnosis of probable or clinically established PD (according to the International Parkinson and Movement Disorder Society criteria), had engaged in the specialized multidisciplinary program for parkinsonian patients at the CNS between January and September 2019, and if they agreed to participate. Exclusion criteria were the inability to adopt a standing position and/or to walk 3 m, postural instability compromising patient safety during the assessment, and the presence of cognitive deficits preventing understanding of the test instructions (according to a physiotherapist's best judgment). The study was undertaken with the understanding and written consent of each participant, with the approval from the CNS Ethics Committee (ref. 10/19), and in compliance with national legislation and the Declaration of Helsinki. Participants were required to agree to all aspects of the study and were able to leave the study at any time.

### Therapeutic Intervention

The specialized multidisciplinary program combined pharmacological and non-pharmacological therapies, including up to 20 h per week of individually tailored neurorehabilitation sessions of physiotherapy, occupational therapy, speech therapy, and cognitive training, according to the patient's needs and rehabilitation goals. All rehabilitation sessions had a duration of 50 min.

The physiotherapy sessions aim to optimize independence, safety, and well-being, through movement rehabilitation, maximization of functionality, and minimization of secondary complications. The sessions focused on physical capacity training, gait, mobility, balance, sensorimotor coordination, and development, as well as teaching the patient and the usual caregivers adaptive strategies to enhance functionality.

### Clinical Assessment Protocol

Patients were assessed in ON-state medication, by a trained health professional from each area, 48 h following admission and before discharge. The following parameters were collected:

- Demographic and clinical data;
- Disease severity: Movement Disorder Society–Unified Parkinson's Disease Rating Scale (MDS-UPDRS) total score and score from each sub-section (8), Hoehn and Yahr scale (8, 9), and Clinical and Patient Global Impression (CGI and PGI, respectively) of Severity and Change; (10)
- Motor function: The Timed Up and Go (TUG) test with and without a cognitive and manual dual-task (5, 11, 12), Mini-BESTest (5, 13, 14), Five times Sit-to-Stand test (5 STS) (15, 16), and Schwab and England scale (17).

### Analysis of Kinematic Data

Kinematic gait parameters were collected during the supervised motor assessments and for 3 days at the end of each assessment, in a free-living (FL) context. Each participant wore a single tri-axial accelerometer-based body-worn monitor (Axivity AX3) on their lower back (L5), programmed to capture raw data at 100 Hz with a dynamic range of ±8 g. Each subject performed two trials of each assessment, on each visit, and wore the AX3 for 3 days after each assessment.

In the supervised motor assessment, the physiotherapist used a mobile application to mark the start and end of each trial, which was synced with the AX3 internal clock. Departing from the segmentation of test trials provided by the application, we manually adjusted the start and end of each test to match with the exact start and end of the movement and removed reported periods of pause. To extract meaningful data from the raw accelerometer signal, we started by resampling data to 100 Hz using linear interpolation, to mitigate known fluctuations of the sample rate (18). Afterward, offset was removed as well as machine noise using a second-order Butterworth low pass filter of 17 Hz (19). We focused the kinematic gait analysis in the study of spatiotemporal gait parameters. To extract gait parameters, the process was divided into two steps. First, we identified the walking bouts as the 2-s moving windows where summed standard deviations of tri-axial accelerations were above 0.1 (20). Then, an algorithm to detect initial contact (IC)/final contact (FC) points was applied, from which we calculated the gait parameters (21). A concurrent validity analysis of the reported number of steps (by the physiotherapist observing the trial) and the automatic detection revealed an intra-class correlation above 0.85.

In the FL context, where walking bouts are not previously annotated, a conservative approach was followed, meaning that high precision was sought (seeking that all detected bouts are indeed bouts), even if at the cost of lower recall (i.e., not all bouts are detected). Pre-processing of FL raw data followed a similar approach as the controlled assessment (resample and filtering). To improve walking bout detection in FL, we estimated an optimized scale of the Gaussian continuous wavelet transform (22) (“gaus2”) and considered only the segments with a duration above 5 s and at least five detected ICs. Additionally, the first and last detected steps of each bout were trimmed off, given their specific transition characteristics. All remaining bouts (and steps) were subjected to extraction of parameters. An average per subject of 285.3 (SD = 175.2, min = 17, max = 622) walking bouts were extracted at the period of admission, and an average of 270.4 (SD = 129.0, min = 32, max = 647) were detected at the period of discharge, in the 3-day period. Gait parameters were calculated from the detected bouts as in the supervised motor assessment (21). Following previously published evidence in FL assessment, gait parameters were categorized in bouts from 5 to 15 s, 15 to 30 s, 30 to 60 s, and longer than 60 s (21). Our implementation of the extraction of gait parameters from walking bouts is available and open-sourced (https://github.com/Gustavo-SF/gait_extractor).

### Statistical Analysis

Descriptive statistics were used for demographic, clinical, and therapeutic data. Continuous outcomes were defined as change from baseline for all the previously mentioned outcome measures and presented as a mean ± standard deviation (SD).

Our main goal was to explore the best predictors of changes in TUG (the gold standard for evaluating FM in PD). To do this, stepwise multiple linear regression analyses were performed using different independent variables (clinical measures, gait parameter assessment during the 10-m walk test, and FL gait parameters analyzed in bouts longer than 60 s). To validate the analysis, the normal distribution of residuals and the absence of multicollinearity were ascertained.

Only the outcome measures able to detect an effect of the intervention were used in the main analysis. This required an assessment, before our main analysis, of the existence of an intervention effect and the ability of the included outcome measures to detect it. We started by studying normality, using the Kolmogorov–Smirnov and the Shapiro–Wilk tests, and applying the paired sample *T*-test and the Wilcoxon S-R test to each parameter to analyze the effects of the program (statistical significance was set at *p* < 0.05). Cohen's *d* was employed as a measure of effect size to assess small (0.20–0.49), medium (0.50–0.80), and large (>0.80) effects (23).

We also performed some exploratory analysis to better understand how the outcome measures, selected as best predictors of FM changes, behave if used as the primary outcome in a future study. Power analysis and sample size calculations were performed using G^*^Power software, to understand how many participants would be needed to enable statistically significant results (80% power) if the TUG test or one of the outcome measures able to detect at least a small effect size were used as the primary outcome in a clinical study. A significance level of α = 0.05 and a power = 1 – β = 0.80 were assumed. To explore the variability of the different gait parameters, a power analysis assuming 10, 20, and 30% of change from baseline and using the mean SD of change from baseline was calculated for each parameter. The choice of the 30% magnitude of effect was based on the minimal clinically important difference (MCID) reported for the TUG test, the recommended measurement tool for assessing FM in PD. It also used a 20% magnitude of effect, based on MCID reported for spatial asymmetry in a previous study evaluating the effect of rehabilitation training on PD patients' gait parameters (25.76%) (24).

Additionally, and also as an exploratory analysis, we applied paired sample *t*-test and the Wilcoxon S-R test to the different bout lengths of FL assessment to investigate how the length of the bout contributes to the existence of a statistically significant difference between admission and the end of the program (significance was achieved with a *p*-value < 0.05).

## Results

### Cohort Demographic and Clinical Data

Of the 54 PD patients who engaged in a CNS specialized multidisciplinary program between January and September 2019, a total of 24 participants were included in this study. The reasons for exclusion were lack of collaboration/missing data (27.8%, *n* = 15), motor inability to perform the assessments (18.5%, *n* = 10), and the presence of cognitive impairment and behavioral disturbances (9.3%, *n* = 5). Eight patients did not perform the FL assessment due to behavioral disturbances and refusal of the belt that supports the trunk sensor. Some of the included patients did not fulfill all the clinical assessment battery due to fatigue and lack of collaboration. The mean age of the participants was 73.0 ± 8.0 years, and 66.7% (*n* = 16) were men. At admission, the average disease duration was 8.0 ± 5.1 years, with a mean Hoehn and Yahr stage of 2.3 ± 0.9 and a mean MDS-UPDRS motor score of 39.4 ± 12.8. All patients were under antiparkinsonian treatment, and 50% (*n* = 12) had motor fluctuations.

Patients' demographic and clinical characteristics at admission and discharge are summarized in Table 1. Table 2 summarizes the changes in gait parameter values in both assessment conditions.

**Table 1:** Demographical and clinical characteristics of the sample.

**Demographic features (*****n*** **=** **24)**				
Age (Mean, SD)	73.04 ± 8.00			
Male sex [% (*n*)]	66.67% (16)			
Body mass index (BMI) (Mean, SD)	25.79 ± 3.90			
Time since diagnosis (Mean, SD)	8.04 ± 5.10			
Presence of motor fluctuations [% (*n*)]	50% (12)			
**Clinical data [Mean (SD), (Range)]**				
	**Admission**	**Discharge**	**Change**	***p*****-value**
MDS-UPDRS I (range 0–52; *n* = 19; ↓)	13.95 ± 7.09	8.25 ± 4.90	−5.53 ± 6.81 (39.6%)	**0.002**
MDS-UPDRS II (range 0–52; *n* = 19; ↓)	17.18 ± 9.24	12.65 ± 7.04	−4.95 ± 10.02 (28.8%)	**0.045**
MDS-UPDRS III (range, 0–132; *n* = 19; ↓)	39.36 ± 12.77	32.20 ± 12.22	−8.52 ± 9.92 (21.7%)	**0.001**
MDS-UPDRS IV (range 0–24; *n* = 19; ↓)	1.95 ± 2.82	1.35 ± 2.16	−0.21 ± 2.53 (10.8%)	0.721
MDS-UPDRS Total (range 0–260; n = 19; ↓)	72.45 ± 25.75	54.45 ± 20.50	−19.26 ± 22.18 (26.6%)	**0.001**
Hoehn and Yahr stage (range 1–5; *n* = 24; ↓)	2.30 ± 0.93	2.35 ± 0.71	0.09 ± 0.68 (3.9%)	0.540
Schwab and England (range 0–100; *n* = 24; ↑)	73.75 ± 16.37	75.83 ± 15.86	2.08 ± 8.33 (2.8%)	0.225
TUG Normal (*n* = 24; ↓)	13.36 ± 7.27	11.68 ± 4.75	−1.69 ± 6.90 (12.7%)	0.243
TUG DT Cognitive (*n* = 23; ↓)	17.22 ± 10.42	14.10 ± 7.29	−2.80 ± 8.91 (16.3%)	0.146
TUG DT Manual (*n* = 19; ↓)	12.80 ± 5.21	11.37 ± 4.35	−0.92 ± 8.69 (7.2%)	0.417
Mini-best (range 0–28; *n* = 19; ↑)	20.19 ± 3.97	20.70 ± 4.59	0.63 ± 3.25 (3.1%)	0.408
5 Sit-to-Stand Normal (*n* = 22; ↓)	19.36 ± 6.99	14.29 ± 5.24	−4.31 ± 2.94 (22.3%)	**0.000**
5 Sit-to-Stand Fast (*n* = 22; ↓)	17.56 ± 4.91	13.25 ± 5.19	−5.07 ± 3.48 (28.9%)	**0.000**
	**Severity (Baseline)**		**Change (Discharge)**	
Clinical Global Impression (*n* = 24; ↓)	4.0 ± 0.83		2.83 ± 0.82	
Patient Global Impression (*n* = 24; ↓)	3.91 ± 1.02		2.50 ± 0.86	

*↑ - a higher score means an improvement, ↓ - a lower score means an improvement. The paired-samples T-test and the Wilcoxon S-R tests were applied to investigate the existence of a statistically significant difference between admission and the end of the program. Significance was achieved with a p-value < 0.05. Bold values is to highlight the outcomes that reached statistical significance*.

**Table 2:** Admission (i.e., baseline) and change from baseline values (i.e., mean post-pre assessment difference and respective percentage value) of gait parameters in the supervised and free-living assessments.

**Gait parameters supervised** ** assessment**	**Supervised assessment**						**Free-living assessment**		
	**TUG normal**			**10-meter walk test**			**Bouts longer than 60 s**		
	**Admission**	**Change from baseline**	***p*-value**	**Admission**	**Change from baseline**	***p*-value**	**Admission**	**Change from baseline**	***p*-value**
Gait velocity (m/s)	0.71 ± 0.19	0.06 ± 0.13 (8.5%)	**0.037**	0.82 ± 0.21	0.05 ± 0.18 (6.1%)	0.188	0.59 ± 0.14	0.04 ± 0.13 (6.8%)	0.209
Cadence (steps/min)	118.77 ± 12.00	1.94 ± 13.02 (1.6%)	0.472	119.92 ± 13.72	3.66 ± 12.95 (3.1%)	0.180	104.93 ± 10.33	−0.92 ± 9.17 (0.9%)	0.695
Stride length (m)	0.78 ± 0.18	0.06 ± 0.14 (7.7%)	0.057	0.89 ± 0.20	0.04 ± 0.17 (4.5%)	0.204	0.69 ± 0.16	0.05 ± 0.13 (7.2%)	0.160
Stride velocity (m/s)	0.71 ± 0.19	0.06 ± 0.13 (8.5%)	**0.033**	0.82 ± 0.21	0.05 ± 0.18 (6.1%)	0.225	0.59 ± 0.14	0.04 ± 0.13 (6.8%)	0.202
Step length (m)	0.39 ± 0.09	0.03 ± 0.07 (7.7%)	**0.049**	0.45 ± 0.10	0.02 ± 0.08 (4.4%)	0.230	0.34 ± 0.08	0.02 ± 0.06 (5.9%)	0.171
Step velocity (m/s)	0.72 ± 0.19	0.06 ± 0.13 (8.3%)	**0.037**	0.82 ± 0.21	0.05 ± 0.18 (6.1%)	0.182	0.60 ± 0.14	0.04 ± 0.13 (6.7%)	0.220
Stance phase (% of gait cycle)	75.26 ± 1.36	−0.11 ± 1.35 (0.2%)	0.708	75.35 ± 0.49	−0.18 ± 1.32 (0.2%)	0.514	75.11 ± 0.55	0.20 ± 0.36 (0.3%)	**0.047**
Swing phase (% of gait cycle)	24.74 ± 1.36	0.11 ± 1.35 (0.5%)	0.708	24.65 ± 0.49	0.18 ± 1.32 (0.7%)	0.514	24.89 ± 0.55	−0.20 ± 0.36 (0.8%)	**0.047**
Double support phase (% of gait cycle)	25.33 ± 1.33	−0.13 ± 1.36 (0.5%)	0.643	25.34 ± 0.51	−0.19 ± 1.27 (0.8%)	0.476	25.11 ± 0.54	0.19 ± 0.36 (0.8%)	**0.050**
Step time (seconds)	0.56 ± 0.06	−0.02 ± 0.06 (3.6%)	0.893	0.55 ± 0.07	−0.01 ± 0.06 (1.8%)	0.525	0.60 ± 0.06	0.002 ± 0.06 (0.3%)	0.896
Stance time (seconds)	0.84 ± 0.09	−0.01 ± 0.09 (1.2%)	0.800	0.83 ± 0.10	−0.01 ± 0.09 (1.2%)	0.589	0.90 ± 0.09	0.004 ± 0.09 (0.4%)	0.845
Swing time (seconds)	0.28 ± 0.04	0.002 ± 0.04 (0.7%)	0.828	0.27 ± 0.03	−0.001 ± 0.03 (3.7%)	0.902	0.30 ± 0.03	−0.001 ± 0.03 (0.3%)	0.930
Double support time (seconds)	0.28 ± 0.03	−0.004 ± 0.03 (1.4%)	0.561	0.28 ± 0.03	−0.004 ± 0.03 (1.4%)	0.916	0.30 ± 0.03	0.004 ± 0.03 (1.3%)	0.583
Stride time variability (% CV)	0.07 ± 0.04	−0.004 ± 0.04 (5.7%)	0.636	0.04 ± 0.02	−0.001 ± 0.03 (2.5%)	0.880	0.12 ± 0.03	−0.01 ± 0.04 (8.3%)	0.393
Step length variability (% CV)	0.05 ± 0.02	−0.003 ± 0.03 (6%)	0.516	0.03 ± 0.01	0.004 ± 0.02 (13.3%)	0.260	0.06 ± 0.01	0.003 ± 0.02 (5%)	0.446
Step time variability (% CV)	0.05 ± 0.03	−0.002 ± 0.03 (4%)	0.730	0.03 ± 0.02	−0.0004 ± 0.02 (1.3%)	0.930	0.09 ± 0.02	−0.01 ± 0.03 (11.1%)	0.210
Step velocity variability (% CV)	0.11 ± 0.04	−0.008 ± 0.04 (7.3%)	0.352	0.06 ± 0.02	0.01 ± 0.04 (16.7%)	0.163	0.13 ± 0.03	0.004 ± 0.03 (3.1%)	0.657
Stance time variability (% CV)	0.06 ± 0.03	−0.005 ± 0.03 (8.3%)	0.384	0.03 ± 0.02	−0.002 ± 0.02 (6.7%)	0.665	0.10 ± 0.03	−0.01 ± 0.03 (10%)	0.340
Swing time variability (% CV)	0.03 ± 0.02	−0.006 ± 0.02 (20%)	0.884	0.02 ± 0.01	0.001 ± 0.02 (20%)	0.862	0.05 ± 0.02	−0.01 ± 0.02 (20%)	0.216
Double support variability (% CV)	0.03 ± 0.02	−0.003 ± 0.02 (10%)	0.455	0.02 ± 0.01	0.00002 ± 0.01 (0.1%)	0.994	0.05 ± 0.02	−0.01 ± 0.02 (20%)	0.163
Stride time asymmetry (% CV)	0.01 ± 0.01	0.002 ± 0.02 (20%)	0.959	0.01 ± 0.01	−0.001 ± 0.01 (10%)	0.584	0.01 ± 0.004	−0.001 ± 0.01 (1%)	0.300
Step time asymmetry (% CV)	0.02 ± 0.02	0.005 ± 0.02 (25%)	0.262	0.03 ± 0.02	0.003 ± 0.02 (10%)	0.496	0.03 ± 0.02	−0.01 ± 0.02(33.3%)	0.318
Stance time asymmetry (% CV)	0.02 ± 0.02	−0.003 ± 0.02 (15%)	0.622	0.02 ± 0.02	−0.003 ± 0.02 (15%)	0.420	0.02 ± 0.01	−0.01 ± 0.02 (50%)	0.153
Swing time asymmetry (% CV)	0.02 ± 0.01	0.008 ± 0.02 (40%)	**0.036**	0.02 ± 0.01	−0.003 ± 0.02 (15%)	0.423	0.02 ± 0.01	−0.01 ± 0.02 (50%)	0.195
Step length asymmetry (% CV)	0.03 ± 0.02	−0.002 ± 0.02 (6.7%)	0.605	0.02 ± 0.02	0.0002 ± 0.02 (1%)	0.959	0.02 ± 0.01	−0.002 ± 0.01 (10%)	0.504

*The paired-samples T-test and the Wilcoxon S-R tests were applied for each parameter to investigate the existence of a statistically significant difference between admission and the end of the program (statistical significance was achieved with p-value < 0.05). Bold values is to highlight the outcomes that reached statistical significance*.

All the clinical and gait parameters from the supervised assessment showed an improvement, having reached statistical significance (*p* ≤ 0.05) in the MDS-UPDRS parts I, II, III, and total score; in the 5 STS test; and the following gait parameters: gait velocity, stride and step velocity, step length, and swing time asymmetry (Tables 1, 2). The improvement in the TUG test did not reach statistical significance, contrary to gait velocity, stride and step velocity, step length, and swing time asymmetry measured during the test. In FL conditions, an improvement was detected when the analysis was made using bouts of at least 30 s. Specifically, the following gait parameters have reached statistical significance (*p* ≤ 0.05): cadence, step time, stance time, swing time, and double support time when data was analyzed in bouts of 30–60 s and stance, swing, and double support phases when bouts of more than 60 s were used in the analysis (Table 2 and Appendix 1).

### Prediction of FM Changes

The stepwise multivariate linear regression analysis, between TUG (dependent variable) and the clinical outcome measures able to detect an effect, indicated the TUG Cognitive as the best variable to predict TUG changes (adjusted *R*^2^ = 0.72). The same analysis using supervised and FL kinematic gait parameters as independent variables identified step length (adjusted *R*^2^ = 0.53) and step time asymmetry (adjusted *R*^2^ = 0.51) as the best predictors of TUG changes for each assessment condition (Table 3).

**Table 3:** Stepwise multiple linear regression analysis with TUG as a dependent variable and (1) the clinical outcome measures, (2) gait parameters assessed during the 10-meter walk test, in supervised conditions, (3) gait parameters assessed in free-living conditions and analyzed in bouts longer than 60 s, as independent variables.

**Dependent variable: TUG change from baseline**	**Predictors**	***R*^**2**^**	**Adjusted *R*^**2**^**	***R*^**2**^ change**	***F***	***p*-value**	**Unstandardized B**	**Standardized coefficients ß**	**Collinearity VIF**
Independent variables: Clinical outcome measures	TUG cognitive	0.75	0.72	0.75	23.59	0.001	0.42	0.86	1.000
Independent variables: Kinematic outcome measures – Supervised assessment	Step length	0.55	0.53	0.55	27.11	0.000	−61.96	−0.74	1.000
Independent variables: Kinematic outcome measures – Free-living assessment	Step time asymmetry	0.55	0.51	0.55	16.79	0.001	104.88	0.74	1.000

### Responsiveness to Intervention

The TUG test was able to detect a small effect size (*d* = −0.24) of the intervention (Appendix 2).

From the supervised assessment, the outcome measures able to detect a large effect size were the STS Normal (*d* = −1.46) and Fast (*d* = −1.47) and the MDS-UPDRS total score (*d* = −0.87).

From the FL assessment, the outcome parameters with higher sensitivity to the intervention were stance time asymmetry (*d* = −0.38), stride length (*d* = 0.37), double support time variability (*d* = −0.37), and step length (*d* = 0.36).

### Sample Size Calculation

A power analysis was performed to understand how many participants would be needed to enable statistically significant results (80% power), if the TUG test or one of the outcome measures able to detect at least a small effect size was used as a primary outcome in a clinical study. Appendix 2 summarizes the sample size calculations assuming 10, 20, and 30% change from baseline.

## Discussion

Although this study was not designed to conclude on efficacy, the results obtained suggest an overall improvement (Tables 1, 2). This enables us to identify the best predictors of FM changes when PD patients are submitted to a specialized multidisciplinary program. It also enables performing other exploratory analyses to better understand how the outcome measures behave if used as primary outcomes in future studies.

From the pool of outcome measures able to detect at least a small effect size of the intervention, those identified as the best predictors of TUG changes were the TUG Cognitive, step length, and step time asymmetry.

### Clinical Assessment

The TUG Cognitive test was the clinical parameter with the best ability to predict TUG changes. This can be explained because the TUG Cognitive is a modified version of the TUG (i.e., it adds a cognitive task to the motor task) (25, 26). Since daily activities frequently require motor and cognitive tasks to be carried out simultaneously, this version of the test may give a more realistic perspective of the patients' FM. However, as it is only a modified version of the same test, some major limitations remain (e.g., it is limited to patients without significant postural instability and is subject to learning effects).

The Mini-BESTest test was not sensitive to the intervention, and the observed differences were not statistically significant. However, this is a very complete clinical test that includes the assessment of static and dynamic balance (i.e., biomechanical constraints, verticality/stability limits, anticipatory postural adjustments, postural responses, sensory orientation, and stability in gait) and the TUG Cognitive test itself (5, 13, 14). Although not formally validated to measure FM, this instrument provides a more complete approach to the three anchors of the concept, i.e., gait, balance, and postural transitions (5, 13, 14). We believe that future studies should clarify the Mini-BESTest's suitability to assess FM changes.

### Clinical vs. Kinematic Assessment

Our results identified step length and step time asymmetry as the gait parameters with the best ability to predict TUG (and FM) changes, in supervised and FL conditions, respectively. Compared with the TUG, both showed higher responsiveness to change.

FM is a major source of disability for PD patients and requires an individualized and complex management approach that strongly depends on the information about the actual state of the patients in their daily lives (1). Although the TUG remains the gold standard for assessing PD FM, as is the case for all traditional clinical scales, it presents some limitations that can be overcome by the use of TOMs (27).

To optimize the accuracy of clinical evaluation, evidence suggests that patients should focus on the goal of the task asked and not on the movement required to achieve it. This is hampered when a reassessment using the TUG test takes place after a multidisciplinary program. During the physiotherapy sessions of the program, patients usually learn safety strategies to apply during walking and postural transitions that require being focused on the movement while doing it. Many of these strategies are applied during the TUG test, thereby hindering its ability to detect an improvement in patients' FM (27).

There is increasing evidence that TOMs may improve the sensitivity, accuracy, reproducibility, and feasibility of data capture, detecting improvements that the clinical tests are not able to find (6). Previous studies reported a greater sensitivity of TOMs, over the traditional clinical scales, in differentiating the gait and turning of PD patients from healthy controls (27).

The use of outcome measures of higher sensitivity and accuracy, which can predict TUG changes (step length and step time asymmetry), may help obtain a more complete and objective evaluation of patients' FM limitations and thereby favoring more personalized clinical decision making (6, 28). In the research field, the use of standardized outcome measures, with high responsiveness to change and low variability, not only enables better interpretation and discussion of research findings but also avoids unnecessary increases in complexity, duration, and financial expenses of studies (6).

Despite the benefits associated with the use of TOMs for assessing FM, from our experience, they also have some limitations. The currently available sensors, although smaller and lighter, remain too intrusive, leading patients to reject their use. Also, in PD patients with behavioral changes, the use of sensors may not be possible. One of the patients was excluded from the FL analysis, after having thrown away the sensors during an episode of delirium.

### Supervised vs. Free-Living Assessment

According to our results, the responsiveness of the outcomes and their ability to predict TUG changes differ depending on the type of assessment.

There is a growing awareness that, depending on the assessment conditions, the results related to gait and postural transitions can differ substantially, with a weak association between the results in both scenarios having been reported (28, 29). Many factors can contribute to these differences: (1) the clear and standardized environment in supervised assessment, in the absence of distractions, emphasizes a measure of someone's best, rather than their usual performance; (2) FL conditions, with narrow corridors, variable lighting, obstacles, etc., forces continuous gait adaptations, inducing large variability and asymmetry in walking patterns; (3) movements in a supervised assessment are triggered by instruction, while FL movements are usually self-initiated, goal-directed, and embedded in a rich behavioral environment; and (4) patients frequently improve their performance when they know that they are being evaluated (21, 28, 29).

In the FL context, gait parameters, and therefore FM, may be influenced not only by physical characteristics but also by ongoing environmental and cognitive challenges (29). Variability and asymmetry-related parameters are especially sensitive to behavioral and environmental factors, better reflecting patients' interaction with the context and their ability to adapt gait patterns (28, 29). We hypothesize that this may be one of the causes of step time asymmetry identified as the FL kinematic gait parameter, which better predicts TUG changes. Although it has only captured a small effect size of the intervention, having a high ecological validity, FL step time asymmetry seems to provide a more realistic picture of the impact of the disease in PD FM, whereby even small changes should be valued (27).

### Length of Walking Bouts

We performed an exploratory analysis to understand how FL gait parameters behave when different bout lengths were used in the analysis. According to our results, there appears to be a link between the ability to capture an improvement and the length of the bout. The longer the walking bouts, the higher the velocity and length of stride/step and the lower the cadence, variability, and asymmetry.

A previous study exploring the impact of environment and bout length in PD patients' gait reached similar conclusions, i.e., the longer the bouts, the higher the increase in step velocity, step length and swing time variability and the lower the variability and asymmetry of gait. The authors also reported that the parameters analyzed in longer bouts were more similar to those measured in a supervised environment (21).

Walking bout length is influenced by the type of environment and activity patients are engaged in (21). Currently, the most suitable length of walking bouts used in FL analysis is not established (21). The majority of studies investigating gait characteristics in FL conditions use bouts longer than 60 s. However, it has been reported that PD patients in FL conditions more often perform a large number of very short bouts (≤10 s) than prolonged bouts (21). According to the literature, bouts of 30–60 s usually represent indoor activities, while bouts > 120 s correspond to walking outdoors. Only bouts with at least 30–60 s were able to discriminate PD patients from healthy controls (21).

### Limitations

This study presents two major limitations: a small sample size (*n* = 24) and high heterogeneity in the included population. We believe that these aspects may overestimate the variability of the measurement tools, influencing the power calculations. We expect that future studies with a large and less heterogeneous population will need a smaller sample size. As an open non-controlled study, we hypothesize that in future larger, controlled trials, the detected effect size will be smaller. However, since this was not an efficacy study (due to the absence of a control group) and an improvement was observed, despite these limitations, we believe that our results are informative and important for the PD field. Also, we believe that the use of broad inclusion criteria in this study not only did not interfere with its aims but also better mimics the real scenario of the intervention and assessments, increasing its external validity. To minimize the impact, the study was conducted in a single tertiary care center.

According to our results, the TUG test did not achieve a statistically significant improvement. However, some of the gait parameters (including step length) not only reached a statistically significant result but also showed a higher sensitivity to change. Since all other results point to an improvement at the end of the program, we believe that this difference may be explained by the greater accuracy and sensitivity to change of TOMs when compared to the traditional clinical scales. A previous study has already highlighted this potential problem, highlighting that the validation of TOMs is often based on their correlation with validated clinical measures and that results may be undesirable, due to the superior capacity of TOMs for capturing the phenomena of interest (30).

## Conclusion

Although we cannot attribute the observed improvements to the specialized multidisciplinary program, our results suggest a methodological approach for identifying outcome measures to assess FM changes, in response to a therapeutic intervention.

From all the outcome measures included in the study, only the TUG Cognitive, step length, and FL step time asymmetry were identified as having the ability to predict TUG changes. The kinematic parameters seem to present higher responsiveness to change when compared with the traditional clinical tests. According to our results, supported by published evidence, the longer the bouts, the higher the sensitivity of detecting an improvement.

Our results support the use of kinematic assessments in evaluating the effect of multidisciplinary interventions in PD FM. The FL step time asymmetry seems a very promising outcome measure to assess FM in PD. Nevertheless, there are some aspects of FL assessments that need to be improved, such as establishing the best data collection protocol and developing less intrusive sensors.

To improve the interpretation of results of responsiveness to change in a complex and fluctuating disease such as PD, it is necessary to clarify the variation of gait parameters in the absence of pharmacological and non-pharmacological therapeutic interventions. This requires repeating the assessment protocol in ON- and OFF-state medication and several times during a short period, thereby clarifying the effect of pharmacological interventions, permitting an understanding of the impact of motor fluctuations and minimizing the interference of disease progression. More studies are also needed to explore the cut-off points from which FM is considered to be affected and the smallest amount of change, in the identified parameters, considered important by the patient or clinician (i.e., the minimal clinically important difference).

## Data Availability Statement

The raw data supporting the conclusions of this article will be made available by the authors, without undue reservation.

## Ethics Statement

The studies involving human participants were reviewed and approved by CNS Ethics Committee. The patients/participants provided their written informed consent to participate in this study. Written informed consent was obtained from the individual(s) for the publication of any potentially identifiable images or data included in this article.

## Author Contributions

RB-M and JF contributed to the conception and design of the study. The CNS physiotherapy study group contributed to the participants' recruitment and assessments. RB-M performed the assessments and performed the statistical analysis. DB, GF, and TG analyzed kinematic data. RB-M and JF drafted the manuscript. All authors contributed to manuscript revision and read and approved the submitted version.

## Collaborators (CNS Physiotherapy Study Group)

Daniela Guerreiro, Verónica Caniça, Francisco Queimado, Pedro Nunes, Alexandra Saúde, Laura Antunes, Joana Alves, Beatriz Santos, Inês Lousada, Maria A. Patriarca, Patrícia Costa, Raquel Nunes e Susana Dias.

## Conflict of Interest

The authors declare that the research was conducted in the absence of any commercial or financial relationships that could be construed as a potential conflict of interest.
