# Supplementary material for: Kinematic and Clinical Outcomes to Evaluate the Efficacy of a Multidisciplinary Intervention on Functional Mobility in Parkinson's Disease
Source: Front Neurol. 2021 Mar 23;12:637620. doi: 10.3389/fneur.2021.637620 (PMC8021905; doi:10.3389/fneur.2021.637620)
Supplement: Supplementary file 1 [file Data_Sheet_1.docx]

**Appendix 1**

Analysis of change from baseline values of gait parameters in free-living according to different bout lengths. The paired-samples T-test and the Wilcoxon S-R tests were applied for each parameter to investigate the existence of a statistically significant difference between admission and the end of the program. Significance was achieved with a p-value<0.05.

| **Gait Parameters**  **Free-living assessment** | **Average** | | **Bouts 15 seconds** | | **Bouts 15-30 seconds** | | **Bouts 30-60 seconds** | | **Bouts longer than 60 seconds** | |
| --- | --- | --- | --- | --- | --- | --- | --- | --- | --- | --- |
|  | **Change** | **p-value** | **Change** | **p-value** | **Change** | **p-value** | **Change** | **p-value** | **Change** | **p-value** |
| Gait Velocity (m/s) | -0.01±0.04 | 0.569 | -0.01±0.04 | 0.288 | -0.02±0.08 | 0.424 | 0.01±0.11 | 0.792 | 0.04±0.13 | 0.209 |
| Cadence (steps/min) | 3.55±7.57 | 0.080 | -3.47±8.82 | 0.137 | -3.06±6.60 | 0.084 | -3.22±4.48 | **0.011** | -0.92±9.17 | 0.695 |
| Stride length (m) | 0.002±0.06 | 0.859 | -0.004±0.06 | 0.809 | -0.005±0.08 | 0.809 | 0.02±0.15 | 0.517 | 0.05±0.13 | 0.160 |
| Stride velocity (m/s) | -0.01±0.05 | 0.591 | -0.01±0.04 | 0.364 | -0.02±0.08 | 0.451 | 0.01±0.11 | 0.754 | 0.04±0.13 | 0.202 |
| Step length (m) | 0.002±0.03 | 0.725 | -0.004±0.03 | 0.642 | -0.004±0.04 | 0.728 | 0.01±0.07 | 0.488 | 0.02±0.06 | 0.171 |
| Step velocity (m/s) | -0.01±0.04 | 0.478 | -0.01±0.04 | 0.281 | -0.02±0.08 | 0.398 | 0.01±0.11 | 0.721 | 0.04±0.13 | 0.220 |
| Stance phase (% of gait cycle) | -0.001±0.40 | 0.995 | -0.09±0.45 | 0.422 | 0.10±0.72 | 0.581 | 0.09±0.39 | 0.395 | 0.20±0.36 | **0.047** |
| Swing phase (% of gait cycle) | 0.001±0.40 | 0.995 | 0.09±0.45 | 0.422 | -0.10±0.72 | 0.581 | -0.09±0.39 | 0.395 | -0.20±0.36 | **0.047** |
| Double support phase (% of gait cycle) | -0.01±0.42 | 0.929 | -0.10±0.47 | 0.387 | 0.09±0.71 | 0.618 | 0.10±0.41 | 0.340 | 0.19±0.36 | **0.050** |
| Step time (seconds) | 0.02±0.04 | 0.096 | 0.01±0.05 | 0.242 | 0.02±0.04 | 0.087 | 0.02±0.03 | **0.011** | 0.002±0.06 | 0.896 |
| Stance time (seconds) | 0.02±0.06 | 0.126 | 0.02±0.07 | 0.293 | 0.03±0.06 | 0.100 | 0.04±0.05 | **0.006** | 0.004±0.09 | 0.845 |
| Swing time (seconds) | 0.01±0.02 | 0.119 | 0.01±0.02 | 0.307 | 0.01±0.02 | 0.264 | 0.01±0.01 | **0.006** | -0.001±0.03 | 0.930 |
| Double support time (seconds) | 0.01±0.02 | 0.159 | 0.01±0.03 | 0.406 | 0.01±0.02 | 0.200 | 0.01±0.02 | **0.010** | 0.004±0.03 | 0.583 |
| Stride time variability (% CV) | -0.004±0.03 | 0.513 | -0.004±0.03 | 0.585 | -0.01±0.04 | 0.389 | -0.01±0.04 | 0.248 | -0.01±0.04 | 0.393 |
| Step length variability (% CV) | 0.0001±0.01 | 0.980 | 0.0004±0.03 | 0.904 | -0.003±0.02 | 0.493 | -0.005±0.02 | 0.227 | 0.003±0.02 | 0.446 |
| Step time variability (% CV) | -0.003±0.02 | 0.473 | -0.004±0.02 | 0.469 | -0.004±0.02 | 0.553 | -0.01±0.03 | 0.294 | -0.01±0.03 | 0.210 |
| Step velocity variability (% CV) | -0.003±0.03 | 0.699 | -0.002±0.03 | 0.840 | -0.01±0.03 | 0.268 | -0.01±0.03 | 0.119 | 0.004±0.03 | 0.657 |
| Stance time variability (% CV) | -0.004±0.02 | 0.480 | -0.004±0.02 | 0.501 | -0.01±0.03 | 0.494 | -0.01±0.04 | 0.186 | -0.01±0.03 | 0.340 |
| Swing time variability (% CV) | -0.005±0.02 | **0.007** | -0.005±0.02 | **0.001** | -0.004±0.02 | 0.413 | -0.01±0.02 | 0.202 | -0.01±0.02 | 0.216 |
| Double support time variability (% CV) | -0.002±0.01 | 0.070 | -0.002±0.02 | 0.244 | -0.004±0.02 | 0.418 | -0.01±0.02 | 0.242 | -0.01±0.02 | 0.163 |
| Stride time asymmetry (% CV) | -0.001±0.004 | 0.284 | -0.002±0.01 | 0.660 | 0.0001±0.004 | 0.881 | -0.001±0.004 | 0.571 | -0.001±0.01 | 0.300 |
| Step time asymmetry (% CV) | -0.001±0.01 | 0.200 | -0.001±0.01 | 0.138 | -0.001±0.02 | 0.903 | -0.003±0.02 | 0.531 | -0.01±0.02 | 0.318 |
| Stance time asymmetry (% CV) | -0.002±0.01 | 0.321 | -0.004±0.01 | 0.573 | 0.002±0.01 | 0.619 | -0.0001±0.01 | 0.977 | -0.01±0.02 | 0.153 |
| Swing time asymmetry (% CV) | -0.003±0.01 | 0.219 | -0.003±0.01 | 0.123 | 0.0001±0.01 | 0.982 | -0.001±0.01 | 0.741 | -0.01±0.02 | 0.195 |
| Step length asymmetry (% CV) | -0.002±0.005 | 0.146 | -0.001±0.01 | 0.174 | -0.005±0.01 | **0.029** | -0.001±0.01 | 0.648 | -0.002±0.01 | 0.504 |
