# Supplementary material for: Kinematic and Clinical Outcomes to Evaluate the Efficacy of a Multidisciplinary Intervention on Functional Mobility in Parkinson's Disease
Source: Front Neurol. 2021 Mar 23;12:637620. doi: 10.3389/fneur.2021.637620 (PMC8021905; doi:10.3389/fneur.2021.637620)
Supplement: Supplementary file 2 [file Data_Sheet_2.docx]

|  | **Cohen’s d** | **Sample size**  (80% power, 30% from baseline) | **Sample size**  (80% power, 20% from baseline) | **Sample size**  (80% power, 10% from baseline) |
| --- | --- | --- | --- | --- |
| **Supervised assessments** |  |  |  |  |
| TUG | -0.24 | 26 | 55 | 211 |
| 5 Sit-to-Stand Fast | -1.47 | 5 | 8 | 22 |
| 5 Sit-to-Stand Normal | -1.46 | 5 | 8 | 23 |
| MDS-UPDRS Total score | -0.87 | 11 | 21 | 76 |
| Stride velocity | 0.46 | 6 | 9 | 30 |
| Gait Velocity | 0.45 | 6 | 9 | 30 |
| Step velocity | 0.45 | 8 | 14 | 47 |
| Swing time asymmetry | 0.45 | 107 | 237 | 941 |
| Step length | 0.42 | 5 | 9 | 27 |
| Stride length | 0.41 | 6 | 9 | 27 |
| TUG Cognitive | -0.31 | 26 | 55 | 213 |
| Schwab and England | 0.25 | 4 | 5 | 13 |
| Step time asymmetry | 0.23 | 131 | 254 | 1575 |
|  |  |  |  |  |
| **Free-living assessment** |  |  |  |  |
| Stance time asymmetry | -0.38 | 54 | 210 | 1302 |
| Stride length | 0.37 | 6 | 9 | 30 |
| Double support time variability | -0.37 | 16 | 45 | 210 |
| Step length | 0.36 | 6 | 8 | 34 |
| Swing time asymmetry | -0.34 | 54 | 210 | 1302 |
| Stride velocity | 0.33 | 7 | 12 | 39 |
| Gait Velocity | 0.33 | 9 | 18 | 63 |
| Step time variability | -0.33 | 16 | 32 | 119 |
| Step velocity | 0.32 | 7 | 12 | 39 |
| Swing time variability | -0.32 | 17 | 34 | 154 |
| Stride time asymmetry | -0.27 | 327 | 1302 | 1302 |
| Step time asymmetry | -0.26 | 67 | 147 | 580 |
| Stance time variability | -0.25 | 16 | 32 | 119 |
| Stride time variability | -0.22 | 16 | 54 | 210 |
| Step length variability | 0.20 | 9 | 16 | 65 |

**Appendix 2** – Clinical and gait parameters able to detect an effect of the intervention (Cohen’s d ≥ 0.20)
